# Proteins secreted by brain arteriolar smooth muscle cells are instructive for neural development

**DOI:** 10.1186/s13041-022-00983-y

**Published:** 2022-11-30

**Authors:** Xuzhao Li, Lili Zhou, Xiaoxuan Zhang, Yuxiao Jin, Bingrui Zhao, Dongdong Zhang, Chengjie Xi, Jiayu Ruan, Zhu Zhu, Jie-Min Jia

**Affiliations:** 1 Fudan University, Shanghai, 200433 China; 2 Key Laboratory of Growth Regulation and Translational Research of Zhejiang Province, School of Life Sciences, Westlake University, Hangzhou, 310024 China; 3 Westlake Laboratory of Life Sciences and Biomedicine, Hangzhou, 310024 China; 4 Laboratory of Neurovascular Biology, Institute of Basic Medical Sciences, Westlake Institute for Advanced Study, Hangzhou, 310024 China; 5 Zhejiang University School of Medicine, Hangzhou, 310058 China; 6 School of Life Sciences, Fudan University, Shanghai, 200438 China; 7 Biotechnology Master’s Program, Brown University, Providence, USA

**Keywords:** Vascular smooth muscle cell (VSMC), Secretome, Neural development, Conditioned medium, Synchronized activity, Neuritogenesis, Neuronal survival, Multielectrode arrays (MEAs)

## Abstract

Intercellular communication between vascular and nerve cells mediated by diffusible proteins has recently emerged as a critical intrinsic program for neural development. However, whether the vascular smooth muscle cell (VSMC) secretome regulates the connectivity of neural circuits remains unknown. Here, we show that conditioned medium from brain VSMC cultures enhances multiple neuronal functions, such as neuritogenesis, neuronal maturation, and survival, thereby improving circuit connectivity. However, protein denaturation by heating compromised these effects. Combined omics analyses of donor VSMC secretomes and recipient neuron transcriptomes revealed that overlapping pathways of extracellular matrix receptor signaling and adhesion molecule integrin binding mediate VSMC-dependent neuronal development. Furthermore, we found that human arterial VSMCs promote neuronal development in multiple ways, including expanding the time window for nascent neurite initiation, increasing neuronal density, and promoting synchronized firing, whereas human umbilical vein VSMCs lack this capability. These in vitro data indicate that brain arteriolar VSMCs may carry direct instructive information for neural development through intercellular communication in vivo*.*

**Supplementary Information:**

The online version contains supplementary material available at 10.1186/s13041-022-00983-y.

## Introduction

Symbiotic communications, for instance, communications mediated by the secretion of diffusible proteins, between the nervous and vascular systems are crucial for brain development [1]. Several neuron-derived signals that modulate the vascularization of the CNS have been described; however, less is known about the vascular signals that orchestrate the development of circuit connectivity in the central nervous system (CNS) [2]. Recent discoveries indicate that beyond the classical role of the vasculature as a conduit to deliver nutrients and oxygen needed for neural activity, vessels serve as a powerful secretory system that controls and instructs a variety of neuronal processes, such as peripheral nervous system (PNS) sympathetic neuron axon targeting and CNS synaptic plasticity [1, 3–7].

Normal neural development begins with appropriate neuronal morphogenesis, during which numerous highly arborized dendrites and long axons are elaborated, thus building the intricate neural circuit network [8]. Neurite initiation, termed neuritogenesis, is the foremost event of neuronal morphogenesis [8]. Failure of nascent formation of neurites leads to neuronal death, encephalopathy, even stillbirth, or mental retardation later in life. Glial cells or neurons are known to be mainly responsible for producing most diffusible molecules to regulate early neural development [9, 10]. However, the direct impacts of the secretome of vascular cells on neuritogenesis, neuronal survival, and functional circuitry establishment remain largely unknown.

Endothelial cells in the inner layer and mural smooth muscle cells (SMCs) or mural pericytes in the outer layer constitute the major building blocks for the entire cerebral vasculature, with SMCs enveloping arteries and arterioles and pericytes covering capillaries. During embryogenesis, VSMCs have a synthetic phenotype, and extracellular matrix production is attributed to their high secretory activity [11]. Synthetic VSMCs gradually switch to a contractile phenotype during vessel maturation, characterized by the expression of more contractile proteins and the reduction of synthetic activity [11]. Previous studies have shown that vascular cells from both layers can secrete axon guidance cues, such as artemin and endothelin, that regulate developing sympathetic neurons in the peripheral nervous system (PNS) [6]. Recent studies have demonstrated that in addition to the PNS, endothelial cells in the CNS produce semaphorin 3G, which directly acts on neuropilin-2/PlexinA4 in neurons to regulate synaptic structure and plasticity [4]. However, since previous studies mostly focused either on developing PNS or on adult CNS synaptic plasticity, the direct effect of vascular cell-secreted proteins on neuritogenesis in the very early stage of neuronal development is not well characterized. In particular, it is unknown whether cerebral arteriolar smooth mural cell-secreted proteins act directly on neurons and accelerate neuron maturation at transcriptomic level, neuronal survival, and circuit development.

Here, we showed that the secretomes obtained from primary mouse cerebral vascular SMCs, a human brain SMC cell line, and a human aorta SMC cell line promote neuronal early morphogenesis, functional maturation, and survival in vitro. In contrast, the human umbilical vein SMC cell line shows detrimental consequences. We used bulk RNA sequencing and mass spectrometry to reveal that the extracellular matrix derived from VSMCs was mostly responsible for these bioactive and promotive activities. Together, these results provide a direct demonstration that ECM factors secreted by the mural smooth muscle of blood vessels function as neuron development supporters by modulating neuritogenesis and protecting neurons from death during early development stages. This study provides new insights into mechanisms underlying vasculo-neuronal coupling–retrograde vascular communication to brain neurons.

## Materials and methods

### Animals

All animal housing, handling, and experimental procedures were conducted in accordance with institutional guidelines, and all animal studies were approved by the Institutional Animal Care and Use Committee (IACUC) of Westlake University, Hangzhou, China. Pregnant mice at mouse embryonic day 18 (E18) and mice at postnatal day 0 (P0) were purchased from the Laboratory Animal Resources Center of Westlake University for all neural cultures.

### VSMC culture and preparation of conditioned media

HBVSMCs, HUVSMCs (ScienCell Research Laboratories, Cat# 1100 and Cat# 8020), and HAVSMCs (ATCC, cat. No CRL-1999) were cultured with smooth muscle cell medium (SMCM) (ScienCell Research Laboratories, Cat# 1101) supplemented with 2% FBS (ScienCell Research Laboratories, Cat# 0010) and 2% smooth muscle cell growth supplement (SMCGS, ScienCell Research Laboratories, Cat# 1152). Penicillin and streptomycin (100 U/mL, Pen Strep, Gibco, Cat# 15140163) were added to the culture medium to prevent bacterial contamination. To collect conditioned media, 90% confluent cultured cells in a T25 cell culture flask were first washed twice with 1X PBS and then fed 15 mL neuronal medium. Conditioned media were harvested after 4 days of conditioning for HBVSMCs, HAVSMCs, and HUVSMCs. Then, the conditioned medium was filtered through a 0.2 µm syringe filter prior to being added to primary neural cultures.

### Mouse hippocampal and cortical neuron culture

Primary hippocampal or cortical neurons from E18 or P0 mouse pups were cultured as described previously with modifications. Briefly, E18 pregnant mice were anesthetized with sodium pentobarbital. Mouse pups (P0) were anesthetized with ice. Ophthalmic forceps were used to quickly dissect the brain tissue and strip the meninges covering the cortex and hippocampus. Next, the hippocampus or cerebral cortex was carefully dissected, cut up, and digested by using 0.125% trypsin for 5–10 min and followed by terminating digestion with 10% FBS. Fifty to one hundred thousand suspended single cells were plated on poly-D-lysine precoated coverslips in 12-well cell culture plates. Hippocampal neurons were cultured in Neurobasal™-A (Gibco, Cat# 10888022), and cortical neurons were in cultured in Neuorbasal medium. Both cultures were supplemented with GlutaMax (GIBCO, 35050-061), B-27™ (Gibco, cat. No 17504044), and penicillin‒streptomycin. Half the volume of the neuronal medium was replaced every three days.

### Immunocytochemistry

Before immunocytochemistry, neurons were first fixed with 4% paraformaldehyde (PFA) for 10–15 min and then washed 3 times with 1X PBS. Then, the neurons were permeabilized with 0.1% Triton-X-100 and blocked with 5% BSA. Mouse anti-TUBB3, phalloidin-647 (Sigma), and rabbit anti-Map2 antibodies were incubated overnight, followed by a 3-h incubation with Alexa Fluor-conjugated secondary antibodies (Invitrogen) at room temperature and counterstaining with Hoechst (1 μg/mL). Finally, coverslips were mounted in Fluorescent G and imaged with a Zeiss 800 confocal laser scanning microscope.

### Longitudinal time-lapse imaging

Live imaging was conducted with an ImageXpress Micro Confocal high-content imaging system (Molecular Device, San Jose). Neural cultures were not transferred to the high-content microscope until 4 h after plating when the cells were well attached to the plate. The interval between two consecutive images was set as 6 min, and neurons were continuously imaged for 36 h.

### Multielectrode array system and recording

One to two days before the isolation of hippocampal neurons, 24-well MEA plates (Axion Biosystems, M384-tMEA-24) were coated with 100 μg/mL poly-d-lysine (Sigma-Aldrich, P0899-50MG) in ddH2O, washed three times with PBS and stored in PBS at 4 °C until use. Prior to use, PBS was aspirated, and plates were dried in a sterilized hood. Dissociated neurons were seeded at a density of 6000 cells/μL in warm Neurobasal-A containing B27, GlutaMax, penicillin/streptomycin, and 5 µg/mL Laminin (Sigma-Aldrich, L2020). Sixty thousand cells were plated on a 24-well MEA plate in a drop with a volume of 10 μL. One hour after plating, an additional 400 μL warm neuron medium or conditioned medium was added to the culture well. Cultures were maintained at 37 °C in 5% CO_2_. Half of the culture medium was changed after each recording session at DIV4, 7, 10, and 14.

MEA recordings were performed as described previously [12] with modifications. Before MEA recordings, plates were equilibrated for 5 min and then recorded for 15 min using an Axion Biosystems Maestro 768 channel amplifier (Axion Biosystems) at 37 °C in a CO_2_ gas-controlled chamber with Axion Integrated Studio (AxIS) software v3.5 (Axion Biosystems). Each well of a 24-well plate comprises 16 electrodes on a 4 × 4 grid, with each electrode detecting the activity of nearby neurons. An adaptive threshold spike detector set at 6× the standard deviation of the noise was used during recordings. Raw data and spike list files were collected. Spike list files were used to extract additional spike, burst, and network features using a custom MEA analysis software package for the interpretation of neuronal activity patterns.

### Flow cytometry analysis (FACS)

Ai14 reporter mice (*tdTomato*^*f/f*^) were crossed with a tamoxifen-inducible SMACreER recombinase driver line to generate *SMACreER:Ai14* double transgenic mice. Leptomeninges of *SMACreER:Ai14* mouse pups at ages P0-6 were dissected and digested with 0.25% trypsin. After a 2-day culture, tamoxifen (5 mM) was added to induce red fluorescent protein tdTomato expression in VSMCs. When the mixed primary pial cells (tdTomato^+^ and tdTomato^−^ cells) became 90% confluent, single-cell suspensions were obtained via 0.25% trypsin digestion and maintained in SMCM. tdTomato^+^ VSMCs were sorted with a LE-MA900FP flow cytometer (SONY, Japan). A 100-μm chip (Cat# LE-C3210) and a PBS sheet fluid pressure of 20 psi were used. First, cells were selected with a very wide gating setting using forward scatter area/side scatter area (FSC-A/SSC-A). Second, based on FSC-A/FSC-H (forward scatter high) and further SSC-A/SSC-H (side scatter high), adherent cells were removed from the parental FSC-A/SSC-A gate. Finally, fluorescent events were selected from the nonadherent cells. tdTomato was excited with a 561-nm laser, and its emission was detected with a 585/30 filter. Cells expressing tdTomato were sorted directly into SMCM and seeded on plates for the following primary culture experiments. All FACS data were analyzed using SONY Cell Sorter software.

### Reverse transcription-PCR

Total RNA from mouse cerebral cortices or sorted primary brain VSMCs was extracted with TRIzol (Sangon Company, Cat# B511311, Shanghai, China). cDNAs were subsequently reverse transcribed using HiScript III RT SuperMix for qPCR + gDNA wiper (Vazyme, Cat# R323-01, Nanjing, China). Each reaction comprised a total volume of 20 μL, containing 1 μL of cDNA, 10 μL of Green Taq Mix (Vazyme, Cat# P131-03, Nanjing, China), each primer pair at 1 μM (Tsingke, Beijing, China) and 7 μL of ddH2O. RT‒PCR products were separated by agarose gel electrophoresis, and images were acquired using a Tanon 2500 (Tanon, Cat# m1956, Shanghai, China). Primer sequences for *Acta2* were as follows: F′, CGGACACGGACAGGATTGACA and R′, CCAGACAAATCGCTCCACCAACTA. Primer sequences for 18S rRNA were as follows: F′, AGCCATCTTTCATTGGGATGG and R′ CCCCTGACAGGACGTTGTTA.

### RNA purification, library preparation, and sequencing

For bulk RNA-seq of cultured neurons, RNA was extracted using TRIzol. Library construction and RNA sequencing were completed by Novogene (Beijing, China).

### RNA-seq data preprocessing, alignment, and transcript abundance quantification

Sequencing data quality checks were performed by using FastQC to check the quality of the generated reads (.fastq files). No files were reported to be of poor sequence quality. Single-end raw reads were trimmed by Trim Galore (version 0.6.7). Then, HISAT2 (version 2.2.21) was used to map the reads to the mouse reference genome (Ensemble 96). The trimmed reads were aligned to the mouse reference genome (GRCm38.p6) with Ensembl annotation (Mus_musculus.GRCm38.96.chr.gtf). featureCounts were used for calculating transcript abundance in units of counts per million (CPM) or per million mapped fragments (FPKM). Unless otherwise specified, the calculations were conducted at the Westlake University high-performance computing center.

### Identification of DEGs

Genes at low abundance (mean CPM < 1 or counts < 10) were excluded prior to downstream analyses. DESeq2 (version 4.2) was used to identify DEGs between neuron groups with or without VSMC-CM treatment from 4 replicates. A false discovery rate (FDR) of < 0.05 and log2-fold change > |1| were used as the criteria to identify differentially expressed genes. All DEG analysis results are summarized in Additional file 5.

### Secretomic analysis by mass spectrometry (LC‒MS/MS)

Twelve secretome preparations were made, six from primary MBVSMCs, four from HBVSMCs and one each from HAVSMC-CM and HUVSMC-CM. The secretomes of HAVSMC and HUVSMC were obtained from HAVSMC and HUVSMC cultured in neurobasal A (B27-free), respectively. Out of the six secretomes from mice, five preparations were from MBVSMCs cultured in SMCM (serum-free) for 4 h and one from cells cultured in neurobasal A (B27-free) for 4 days. Out of the four preparations from HBVMSCs, two were in SMCM (serum-free), and two were in neurobasal A with B27 or without B27 in each preparation. Because B27 contains BSA, the fractions falling within 50–70 kD were removed after running the separating SDS–PAGE gel. Two independent methods were used for secretome extraction or enrichment from the conditioned medium (VSMC-CM). One method was as follows: VSMC-CMs were concentrated with a 10-kDa molecular mass cutoff membrane (Millipore, Billerica, MA, USA) by centrifugation at 2000*g* at 4 °C until the volume of media reached 100 µL. The other method involved lyophilizing VSMC-CMs to dryness and desalting them using an NAP5 column (GE Healthcare). SDS–PAGE gels were used to remove the detergent from the protein sample. The SDS–PAGE gel containing the protein sample band was cut and digested with trypsin prior to reduction and alkylation in 50 mM ammonium bicarbonate at 37 °C overnight. The digested products were extracted twice with 1% formic acid in 50% acetonitrile aqueous solution and dried with a speedvac to reduce the volume. SDS‒PAGE was used to separate proteins, which were stained with Coomassie Blue G-250.

For LC‒MS/MS analysis, the peptides were separated by 65-min gradient elution at a flow rate of 0.300 µL/min by using the Thermo EASY-nLC1200 integrated nano-HPLC system, which was directly interfaced with the Thermo Q Exactive HF-X mass spectrometer. Home-made analytical columns were fused with silica capillary columns (75 µm ID, 150 mm length; Upchurch, Oak Harbor, WA) and packed with C-18 resin (300 A, 3 µm, Varian, Lexington, MA). Mobile phase A consisted of 0.1% formic acid in water, and mobile phase B consisted of 100% acetonitrile and 0.1% formic acid. Xcalibur 4.1 software was used to operate the mass spectrometer in the data-dependent acquisition mode. A single full-scan mass spectrum in the Orbitrap (400–1800 m/z, 60,000 resolution) was followed by 20 data-dependent MS/MS scans at 30% normalized collision energy. The AGC target was set as 5e4, and the maximum injection time was 50 ms. Each mass spectrum was analyzed using the Thermo Xcalibur Qual Browser and Proteome Discovery for database searching and label-free analysis.

### Functional annotation and gene set enrichment

Kyoto Encyclopedia of Genes and Genomes (KEGG) and Gene Ontology (GO) enrichment pathways were analyzed using the clusterProfiler (version 4.2) R package to obtain statistically enriched terms from the following categories: cellular component (CC), molecular function (MF), and biological process (BP). A cutoff P-adjusted value of < 0.05 was considered to indicate significant enrichment.

### Protein interaction analysis

Protein interaction analyses were conducted in the web of STRING. Protein encoding genes in ECM-receptor interaction pathway, the common signaling pathway between neuron transcriptome and VSMC-CM secretome, were selected and conducted the analyses with two modes: The experimentally confirmed mode and the database prediction mode. Protein interaction network was shown and the line thickness between two proteins indicated the strength of data support.

### Statistical analysis and data availability

For the analyses of neurite numbers, only neurite outgrowth from the neuron soma was counted. The Neuron J plugin in ImageJ was used to measure neurite length and conduct neuronal Sholl analysis. The distribution curve of neurite number and the cumulative curve were presented by using Minitab 19 and GraphPad Prism 7 software, respectively. To calculate the statistical significance of differences in neurite number, neurite length, and neuron density, a two-tailed, unpaired Student’s t test in GraphPad Prism 7 software was performed. For statistical analysis of MEA data, to compare more than two groups, one-way analysis of variance (ANOVA) with Dunnett’s post hoc test was used. Statistical significance is expressed as follows: *P < 0.05; **P < 0.01; ***P < 0.001. Data in graphs are presented as the mean ± S.E.M. or the mean ± SD.

## Results

### Conditioned medium from human and mouse brain vascular SMCs promotes the initial steps of neural morphogenesis

**Fig. 1:**
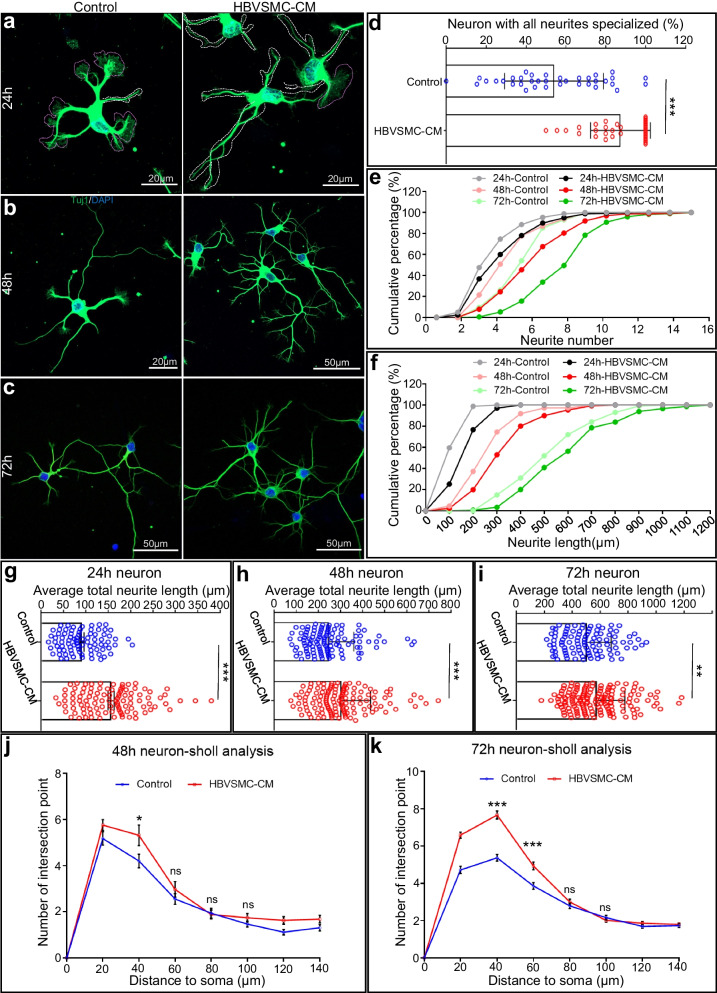
HBVSMC-CM promotes neuron development by accelerating single neurite maturation and increasing neurite numbers. **a**–**c** Representative images of hippocampal neurons stained with an anti-Tuj1 antibody (green) and counterstained with Hoechst (blue). Specialized neurites are outlined with white dashed lines, and non-specialized neurites with large growth cones are outlined with magenta dashed lines. **d** Quantification of neurons in which all neurites were specialized in the control and HBVSMC-CM groups. **e**, **f** Cumulative curve of the percentages of neurons with different neurite numbers (**e**) and neurite lengths (**f**) at 24, 48, and 72 h. **g**–**i** Quantification of average total neurite length per neuron at 24 (**g**), 48 (**h**), 72 h (**i**). **j**, **k** Sholl-analysis of 48-h-old (**j**) and 72-h-old (**k**) hippocampal neurons with or without HBVSMC-CM treatment

Hippocampal neurons undergo five consecutive stages of stereotypical development, and most of them complete stage 2 of neuritogenesis within 24 h. Newly formed neurites develop into the axons or dendrites of mature neurons, forming the intricate circuitry throughout the entire nervous system. To examine whether vascular SMCs are involved in neuritogenesis and subsequent dendrite development, we first collected conditioned medium from a human brain vascular smooth muscle cell line (HBVSMCs) (Additional file 1: Fig. S1a). We cultured HBVSMCs for four days using neural basal A and obtained conditioned medium (HBVSMC-CM) with normal pH levels and osmolarity compared to those of fresh medium (Additional file 1: Fig. S1b, c and Additional file 2: Table S1). We added HBVSMC-CM to dissociated hippocampal neurons 4 h after plating and examined their morphological changes at time points of 24, 48, and 72 h by immunocytochemistry with antibodies against the neuronal marker Tuj1 (Fig. 1) and the dendrite marker MAP2 (Additional file 3: Fig. S2).

First, we measured nascent neurite development 24 h after plating, a time window in which active neuritogenesis and neuronal polarity establishment occur [13]. None-specialized neurites refer to neurites with a large growth cone at their terminal tips, as outlined by magenta dashed lines in Fig. 1a, and specialized neurites with tapered endings are outlined by white dashed lines in Fig. 1a. At this stage, one neuron possesses multiple neurites, which can be specialized, non-specialized, or mixed. We found that the number of neurons with all neurites specialized was higher than that of untreated neurons (87.39% mean ± 2.575 SE vs. 45.98% mean ± 4.13 SE, Fig. 1d). Neuritogenesis takes place throughout the early stages of these cells' growth (before synapse formation). We thus conclude that molecules secreted by HBVSMCs accelerate neurite development, which occurs independently of synapse formation. Additionally, the development of brain networks depends on neurite maturation. Our findings suggest a potential role for local cerebral VSMCs in promoting brain network development.

In addition, we compared the total number of nascent neurites directly extending from the neuronal soma and subsequently developing into single axons or primary dendrites [8]. We found that at all time points, neurons treated with HBVSMC-CM showed more primary neurites than untreated cells, indicating positive changes in neuronal development (Fig. 1e, light color vs. heavy color). Notably, the neurite number of the HBVSMC-CM-treated neurons at 48 h (red curve) (Control: 4.67 ± 1.66, HBVSMC-CM: 5.97 ± 2.13) even surpassed that of the control neurons at 72 h (light green curve) (Control: 5.33 ± 1.41, HBVSMC-CM: 7.69 ± 2.16), indicating that brain VSMCs accelerate early neuronal development and suggesting that the presence of VSMCs in the brain microenvironment finely tunes the rate of neuron development. Meanwhile, we found increased averaged total neurite length per neuron in the treated groups at 24 h (Fig. 1g, Control: 91.18 ± 4.78 µm vs. HBVSMC-CM: 87.39 ± 2.575 µm), 48 h (Fig. 1h, Control: 247.2 ± 10.83 µm vs. HBVSMC-CM: 304.4 ± 11.59 µm) and 72 h (Fig. 1i, Control: 505.7 ± 17.44 µm vs. HBVSMC-CM: 578 ± 16.32 µm). Cumulative percentage analysis of total neurite length revealed the same promotion effects (Fig. 1f), while we did not observe the surpassing phenomenon as did neurite number (Fig. 1e). These results demonstrated that HBVSMC-CM not only accelerated the transformation of individual non-specialized neurites to specialized neurites but also increased primary neurite formation and neurite outgrowth.

Following nascent neurite initiation, most primary neurites become primary dendrites during neuronal polarity establishment [14], during which multiple minor dendrite branches subsequently form a complex dendritic arbor of CNS neurons. Sholl analysis revealed more intersections in the proximal region of the soma but comparable crossing points at more distal sites (Fig. 1j, k). The dendrite-specific marker MAP2 (red) was utilized to stain neurons to corroborate this finding, and the results are compatible with those of the analysis performed using a Tuj1 (green) antibody (Additional file 3: Fig. S2). Taken together, these data indicated that subsequent dendrite branching in the HB-VSMC-treated group was likely augmented as well.

To test whether primary mouse brain VSMCs (MBVSMCs) have effects similar to those of HBVSMCs. We cultured sorted MBVSMCs from *SMACreER:Ai14* mice with dual genetic modifications, in which VSMCs were labeled by inducible tdTomato expression under the control of the SMA promoter [15]. The purity of cultured MBVSMCs reached 99% (Additional file 4: Fig. S3a, b). The RT‒PCR results revealed a 17-fold higher mRNA expression level of alpha-smooth muscle actin (a-SMA, a marker of SMCs) in the sorted MBSMCs than in the cerebral cortex tissue (Additional file 4: Fig. S3c), further demonstrating the high purity of the primary MBVSMC culture that we established.

**Fig. 2:**
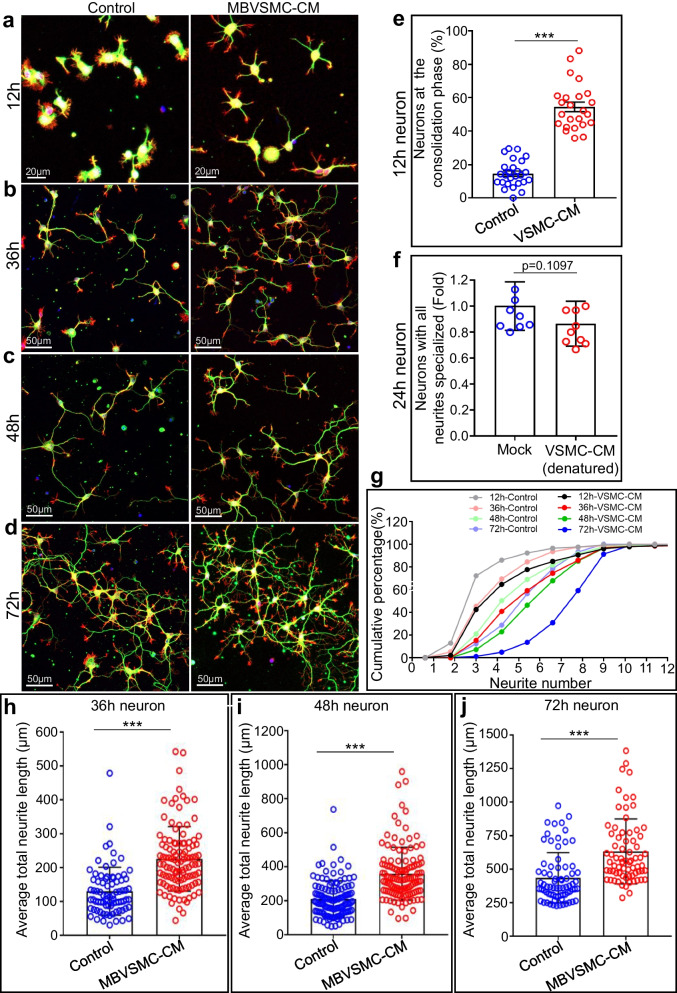
The secretome of mouse brain VSMCs promotes neuron development. **a**–**d** Representative images of hippocampal neurons immunostained with an anti-Tuj1 antibody (green) and stained with phalloidin-647 (red) after culture for 12 (**a**), 36 (**b**), 48 (**c**), and 72 (**d**) hours. **e** Quantification of neurons entering the consolidation phase after culture for 12 h. **f** Fold changes in neurons in which all neurites were in a specialized form between the two groups with or without denatured VSMC-CM. **g** Cumulative curve of the percentages of neurons with different neurite numbers after treatment with MBVSMC-CM at 12, 36, 48, and 72 h after plating. **h**–**j** Quantification of average total neurite length per neuron after cultured for 36 (**h**), 48 (**i**), 72 h (**j**)

After being plated in culture, spherical neurons initially start projecting circumferential lamellipodia within a short period of time (0–6 h, stage 1); these lamellipodia eventually protrude forward and engorge to produce cylindrical neurites, known as the consolidation phase (6–24 h, stage 2). After mouse hippocampal neurons were treated with MBVSMC-CM and immunostained with an anti-Tuj1 antibody and co-stained with the actin-binding reagent phalloidin, we found that at the 12-h time point after plating, more than half of neurons that were exposed to MBVSMC-CM proceeded to the consolidation phase, while less than 20% of the untreated neurons proceeded to the consolidation phase (Fig. 2a, e). As a control, we treated neurons with the conditioned medium that had been subjected to heating, which denatured proteins. The neurite counts then remained unchanged compared to those of control neurons (Fig. 2f). These results together indicate that the secreted proteins (the secretome) from MBVSMCs display bioactivity by promoting earlier morphogenesis of transitioning lamellipodium to nascent neurites, in addition of promoting neurite maturation.

Cumulative analyses confirmed the findings from HBVSMC-CM, showing that younger MBSMC-CM-treated neurons had more neurites than the older control neurons (12 h-CM (4.33 ± 2.26) vs. 36 h-Control (3.86 ± 1.72), 36 h-CM (5.36 ± 2.04) vs. 48 h-Control (4.91 ± 1.85), and 48 h-CM (5.85 ± 1.82) vs. 72 h-Control) (5.29 ± 1.52) (Fig. 2g). Similarly, the averaged total neurite length per neuron is longer in the treated neurons than in control neurons (Fig. 2h–j) (36 h-Control: 130.3 ± 70.83, 36 h-MBVSMC-CM: 225.9 ± 95.57, 48 h-Control: 211.0 ± 106.0, 48 h-MBVSMC-CM: 359.7 ± 155.4, 72 h-Control: 436.4 ± 187.8, 72 h-MBVSMC-CM: 633.4 ± 242.6). The conditioned media utilized in the subsequent investigations, which were all conducted using mouse primary cells or human cell lines, is referred to as brain VSMC-CM. These results revealed that unexpected effects of brain VSMCs on neuron development are conserved from mouse to human cells.

In summary, we demonstrated that the secretome of brain-derived vascular smooth muscle cells accelerates individual neurite specialization and increases initial neuritogenesis and subsequent minor branch formation, which are critical developmental steps for later neural network establishment.

### VSMC-CM expanded the neurite initiation time window from developmental stage 2 to stage 3

The production of neurites in treated neurons may be due to faster neurite generation within the same time period (velocity), a longer time window, or both. To investigate this issue, by using high-content microscopy, we conducted longitudinal time-lapse imaging for 36 consecutive hours, starting 4 h after neuronal plating.

**Fig. 3:**
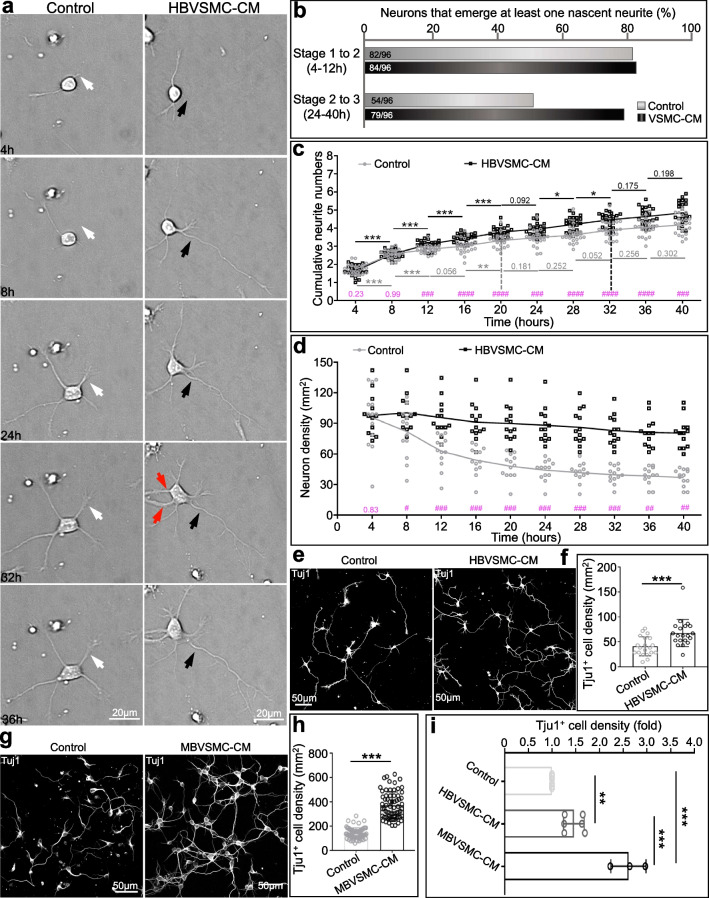
The HBVSMC secretome expands the time window of neurite initiation and protects against neuronal death. **a** Images of hippocampal neurons that were initiating nascent neurites. White and black arrows indicate neurite initiations that occurred by 24 h after plating. Red arrows point to neurite initiations that were generated 24–40 h after plating. **b** Quantification of the percentage of neurons that possessed the ability to generate nascent neurites. **c** Cumulative neurite numbers across the entire imaging period. P values in black and gray refer to statistical comparisons between two consecutive time points in the same group, while P values in magenta refer to statistically significant differences between two groups at the same time point. **d** Quantitative analysis of neuron density over time. **e** Representative images of neurons treated with or without HBVSMC-CM that immunostained with an anti-Tuj1 antibody. **f** Quantification of Tuj1^+^ cell density in **e**. **g** Representative images of neurons treated with or without MBVSMC-CM that immunostained with an anti-Tuj1 antibody. **h** Quantification of Tuj1^+^ cell density in **g**. **i** Fold change analyses of Tuj1^+^ cell density in HBVSMC-CM and MBVSMC-CM compared with that in control group

First, we found that comparable proportions of cells in both conditions (Control: 82 cells out of 96, 85.5%, compared to HBVSMC-CM: 84 cells out of 96, 87.5%) had the ability to generate at least one new neurite within the 4–12 h time window (white arrow and black arrow, Fig. 3a, b). Notably, the majority of treated neurons maintained this ability over the subsequent 24–40 h, but control neurons did not (Control: 56.3% vs. HBVSMC-CM: 83%, red arrows, Fig. 3b). The time course of 4-h bin analysis showed that the neuritogenesis time window was 12 h longer for HBVSMC-CM-treated neurons than for control neurons (time difference between the gray vertical dash line and the black vertical dash line) (Fig. 3c). Control neurons stopped generating nascent neurite 20 h after plating, whereas the treated neurons did not stop generating nascent neurite until 32 h after plating. Although the neuritogenic activity was comparable between the two treatments during the first 4 to 8 h (P = 0.23 at 4 h and 0.99 at 8 h, pink, Fig. 3c), this activity of treated neurons increased significantly at 12 h after plating (^###^P < 0.001, pink, Fig. 3c). This result demonstrated that HBVSMC-CM treatment not only expanded the time window but also led to faster neurite growth (Fig. 3c).

### VSMC-CM increases the primary neuron number in developing neural cultures

In addition to neuritogenesis processes, longitudinal time-lapse imaging revealed HBVSMC-CM to be neuroprotective in the relatively stressful environment within a high-content microscope with more unstable culture conditions than a standard incubator. We analyzed the number of neurons in each group in 12 regions of interest (ROIs) of the same area at 11 different time points separately (Fig. 3d). We noted that although the number of neurons was the same at the initial 4-h time point, there was an initial sharp drop in the number of neurons during the first 8 culturing hours (from 4 to 12 h). This is the time point when neuronal death usually occurs during the early period of in vitro culture. Interestingly, we did not observe such a decline in HBVSMC-CM-treated neurons (Fig. 3d). Multiple independent experiments that used anti-Tuj1 antibodies to determine neuron numbers revealed that although HBVSMC and MBVSMC had similar promotion effects at 48 h (Fig. 3e–h), the effect of MBVSMC-CM has stronger potency than that of HBVSMC-CM (Fig. 3i).

Together, these data demonstrated unexpected neurotrophic functions of VSMC-secreted proteins, such as promoting neurite specialization, extending the neurite initiation time window, and protecting neurons from death under mild stress, suggesting that VSMC dysfunction depends in part on their secretory function. Under normal physiological conditions where vasomotor activity is well maintained, dysfunction of secretion in VSMCs may affect brain development.

### Identification of DEGs between VSMC-CM-treated and control neurons

**Fig. 4:**
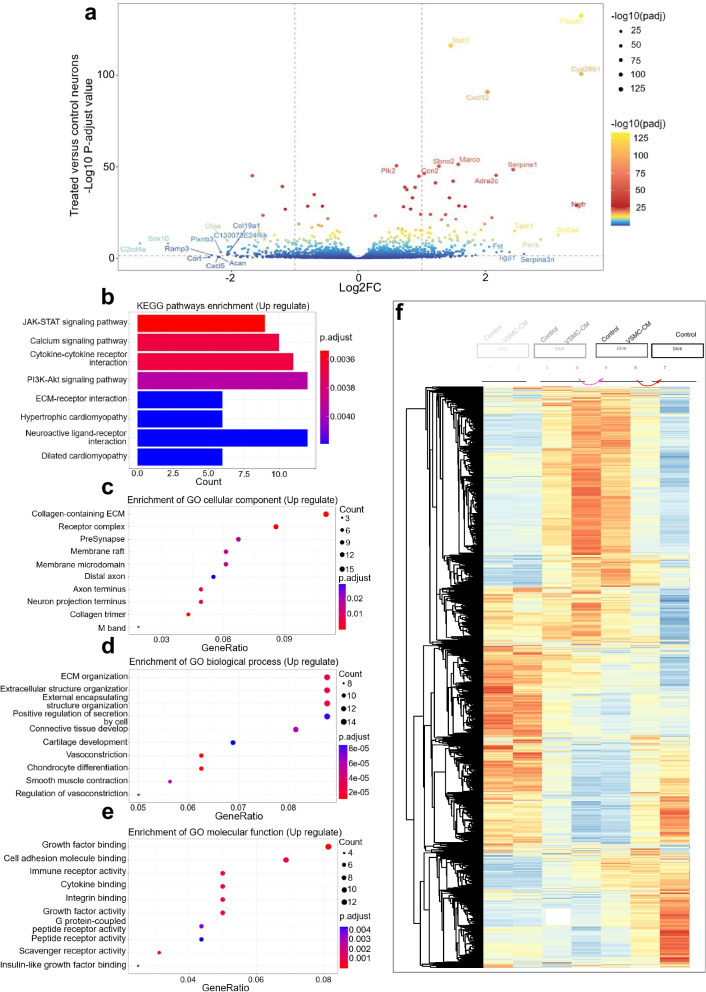
RNA-Seq transcriptomic analysis of primary cortical neurons treated with VSMC-CM. **a** Volcano plot of DEGs identified by comparing HBVSMC-CM-treated neurons with control neurons. A log2-fold change on the x-axis ≥ |1|  and log2 False Discovery Rate < 0.05 were considered statistically significant. **b** KEGG analysis of significantly upregulated genes in neurons treated with VSMC-CM. The ordinate is KEGG signaling pathways, and the abscissa is the number of genes of pathways. **c**–**e** Subsets of categorized GO terms of upregulated genes identified in **b**. The top 10 significantly enriched GO terms for Cellular Component (**d**), Biological Process (**c**), and Molecular Function (**e**) were selected by a false discovery rate (FDR) < 0.05. **f** Heatmap of neuronal transcriptomes at different days in vitro (DIV). Heatmap of neuron total genes with CMP value at least > 1, with hierarchical clustering based on the average cluster method. Lanes 1 and 2 indicate the DIV2 control and treated groups, lanes 3 and 4 indicate the DIV4 control and treated groups, lanes 5 and 6 indicate the DIV6 control and treated groups, and lane 7 indicates the DIV 8 control group

To gain molecular insight into neurons responding to the VSMC secretome, we performed high-throughput gene expression profiling to identify biomolecular processes in the recipient neurons at day 4 in vitro (DIV 4) to unveil transcriptomic differences between neurons under the two conditions. We identified a total of 427 genes that were differentially expressed in HBVSMC-CM-treated neurons compared to control neurons, with a P-adjusted threshold < 0.05 (Fig. 4a). Among these differentially expressed genes (DEGs) (Additional file 5: Table S2), we identified 260 upregulated genes in HBVSMC-CM-treated neurons, including *Ngfr, Fibcd1, Cyp26b1, Slc6a4, and Serpine1.* All these genes are commonly involved in neuron growth, survival, and central nervous system development, with damage to some of these genes resulting in impaired neurodevelopment or delayed neuronal maturation [16–23]. In contrast, genes that inhibit neuron maturation were downregulated after neuronal exposure to VSMC-CM, such as *Sox10,* which is a well-established transcription factor that inhibits neuron development [24] (Fig. 4a), suggesting that HBVSMC-CM selectively upregulates genes that promote neural development and cell survival on the one hand and represses the maturation inhibitory program on the other. Of the first eight pathways enriched by KEGG analysis of upregulated genes, five were mapped to pathways involved in cell growth or survival, including “JAK-STAT signaling pathway, calcium signaling pathway, cytokine‒cytokine receptor interaction, PI3K-Akt signaling pathway, ECM-receptor interaction, and neuroactive ligand‒receptor interaction” (Fig. 4b). These pathways are commonly involved in regulating neurogenesis, neuritogenesis, neurite outgrowth, and cell survival processes [25–29]. There was no obvious enrichment of downregulated gene pathways.

Having identified DEGs and their KEGG signaling pathway annotations, we next investigated whether the upregulated genes were significantly enriched in gene ontology (GO) terms. The top enriched subontologies of ‘cellular component’, ‘molecular function’, and ‘biological process’ reflected the involvement of growth factor binding and cell adhesion molecular binding in the extracellular matrix in the regulation of neuronal extracellular organization and enhanced outside-in signaling transduction in neurons exposed to VSMC-CM (Fig. 4c–e). Consistent with our morphological observations, these findings suggested that VSMC-CM does tune gene profiles in developing neurons, which may positively regulate neuron development and survival, likely through regulation of neuronal ECM organization.

### The VSMC secretome accelerates developmental transcriptomic shifting

To directly assess whether the VSMC secretome promotes neuron maturation at the transcriptional regulation level, we performed bulk RNA-seq of developing neurons at DIV 2, 4, 6, and 8 under both conditions. The unsupervised hierarchical clustering analysis shown in the heatmap demonstrated that the gene expression profile of the treated neurons at DIV2 mimicked that of the corresponding two-day-older control neurons (DIV 4). Similarly, the treated neurons at DIV 4 and DIV 6 mimicked that of DIV 6 and DIV 8 control neurons respectively (Fig. 4f). These findings provide direct evidence that the secretome of VSMCs accelerates neuron development by facilitating transcriptomic shifts toward a mature status.

### Proteomic analysis indicated that VSMC secretome contains signaling proteins that are primarily involved in the extracellular matrix

**Fig. 5:**
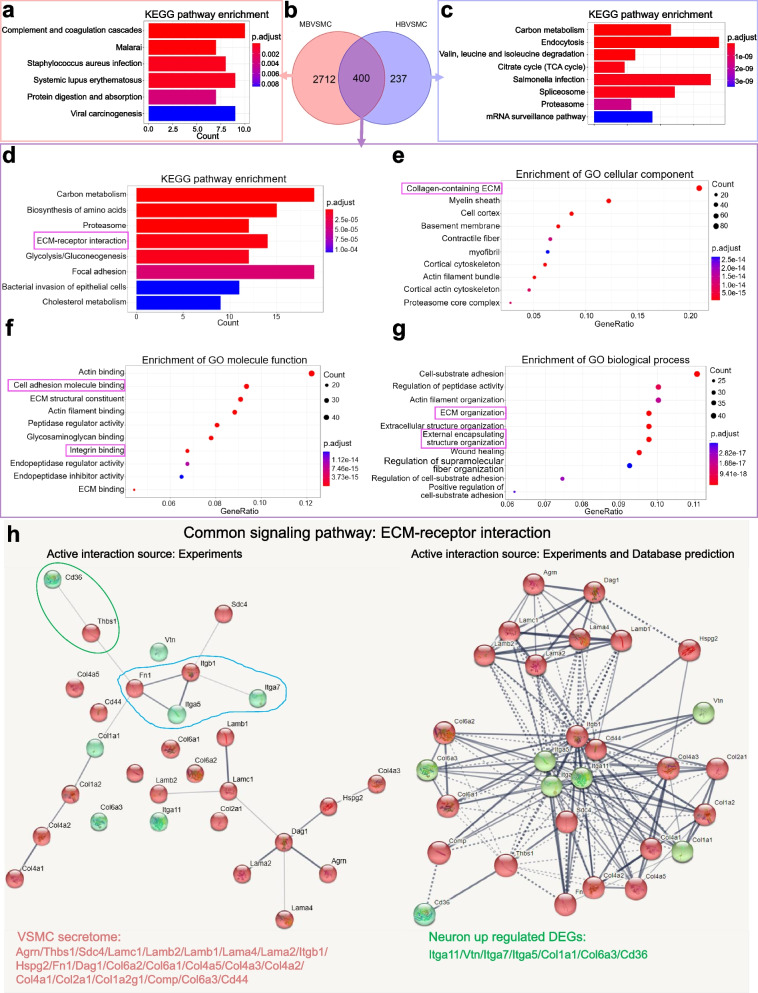
Proteomic analysis of secreted proteins from mouse and human VSMCs. **a** KEGG pathways that were enriched after analysis 2712 MBVSMC-CM specific proteins. **b** Venn diagram of common and exclusive secreted proteins in MBVSMCs and HBVSMCs. Proteins with a Sum PEP Score > 1 were chosen. **c** KEGG pathways that were enriched after analysis 237 HBVSMC-CM specific proteins. **d** KEGG pathways that were enriched after analysis of 400 common proteins. **e**–**g** GO analysis of cellular components (**e**), molecular function (**f**) and biological processes (**g**) for 400 genes. The top ten enriched GO terms for each cluster are shown. **h** Protein interaction analyses of the common signaling pathway with experimentally confirmed mode (Left panel) and database prediction mode (Right panel). The ball color showed the proteins encoding genes come from different collections: VSMC-CM secretome collections (Red) and neuron transcriptome collections (Green). Line thickness indicated the strength of data support

We investigated the proteomes of conditioned media from both cultures of MBVSMCs and HBVSMCs to investigate the possibility that secreted proteins from donor cells may be engaged in intercellular signaling capability during circuit formation in recipient neuronal cultures. We detected a total of 3349 proteins from MBVMSC- and HBVSMC-conditioned media in 3 independent mass spectrometry experiments. Venn diagram analysis showed 2712 proteins exclusively detected in MBVSMC-CM, 237 proteins in HBVSMC-CM and a total of 400 proteins commonly identified in both mouse and human cell samples (Fig. 5b, 400 common protein names listed in Additional file 6: Table S3). KEGG enrichment analyses for the differential proteins revealed no overlapped enriched pathways (Fig. 5a, c). The top enrichment pathways suggested that different type of VSMCs may have distinct functions. In contrast, when we further used these 400 genes for the following KEGG and GO term analyses we identified overlapped pathways (Fig. 5d–g). We compared the pathways derived from donor brain VSMC secretome proteomic data with those derived from recipient developmental neuron transcriptome data. Notably, we found that these two sets of omics data coincide with each other by focusing on the following pathways: (1) “ECM-receptor interaction” in KEGG analysis, (2) “collagen-containing extracellular matrix” in the cellular component category, (3) “cell adhesion molecule binding” and “integrin binding” in the molecular function category, and (4) extracellular matrix organization” and “external encapsulating structure organization” in the biological process category (highlighted in the magenta box, Fig. 5d–g). Among these convergent terms, the extracellular matrix signal appeared to be the central pathway mediating all the aforementioned neural functional outcomes. To reveal the potential ECM ligan-receptor pairs, we further performed protein–protein interaction networks functional enrichment analysis by using STRING (Search Tool for the Retrieval of Interacting Genes/Proteins). We found three ligand-receptor pairs (*Thbs1*-*Cd36*, *Fn1*-*Itga5,* and *Itgb1*-*Itga7*, circled in green and blue in Fig. 5h) that were identified in both the experimentally confirmed mode and database prediction mode (Fig. 5h). Interestingly, among these pairs, *Thbs1*, which encodes thrombospondin 1, a well-documented immature astrocyte-secreted synaptogenic factor [30, 31], was secreted by VMSCs as well. Thus, our results demonstrated VSMC is another important cell source for secreting thrombospondin 1 in the brain. These findings may provide hints that vascular smooth muscle cells may regulate brain development by secreting extracellular matrix proteins that reorganize the external structure of neurons.

### Different organ-derived VSMCs have distinct effects

**Fig. 6:**
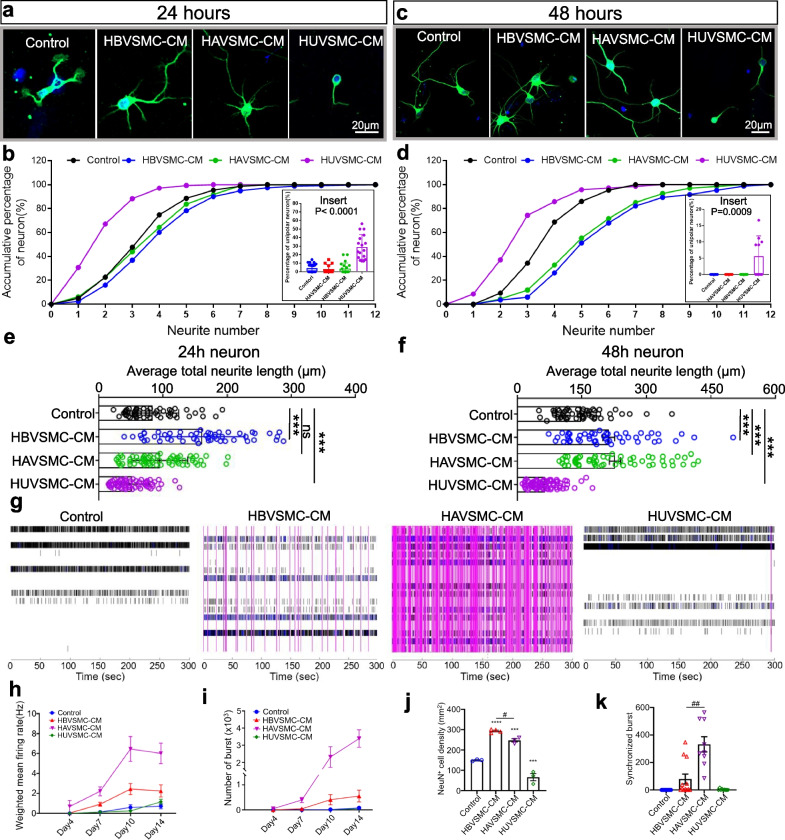
Different organ-derived VSMC cell lines have diverse effects on neural development. **a** Representative images of hippocampal neurons treated with HAVSMC-CM, HBVSMC-CM, and HUVSMC-CM for 20 h starting at 4 h after plating. anti-Tuj1 (green), Hoechst (blue). **b** Cumulative probability of neurons with different neurite numbers. Insert: percentages of unipolar neurons in the whole population of neurons calculated from the same culture. **c** Representative images of hippocampal neurons with the same treatments as **a** with an additional 44 h after plating. **d** Cumulative probability in **c**. Insert: percentages of unipolar neurons in different group. **e**, **f** Quantification of average total neurite length per neuron in different group after treated for 20 h (**e**) and 44 h (**f**). **g** Raster plots generated from MEA measurements of primary hippocampal neurons at DIV14 with neurobasal A medium (control) or HBVSMC-, HAVSMC- and HUVSMC-derived conditioned media. Each black line represents a spike that crossed the noise threshold (± 6 × standard deviation). Blue lines represent a single-electrode burst—a collection of at least 5 spikes within an interspike interval (ISI) of 100 ms. Synchronized bursts are marked with purple boxes and defined as a collection of at least 10 spikes from a minimum of 25% of participating electrodes across the entire well, each separated by an ISI within 100 ms. Each plot represents one well from each control or treatment group during 300 s of recording. **h**, **i** Line charts showing parameters of neuronal functions, including the number of spikes (**h**) and weighted mean firing rate (**i**). MEA recordings were collected at DIV4, 7, 10, and 14 of primary hippocampal neuron cultures and analyzed using 10 min of recordings (Axion Biosystems). **j** NeuN^+^ cell densities of each group were compared at DIV21. One-way ANOVA with Dunnett’s post hoc test was used for statistical analysis. Mean ± SEM. **k** Bar charts exhibiting synchronized bursts at DIV14. Mean ± SEM. Three independent experiments were performed. Data are shown as the mean ± SEM. ***P < 0.001, ****P < 0.0001, ^##^P < 0.01

To investigate whether the neuritogenic and neuroprotective bioactivity of the VSMC secretome is specific to brain-derived VSMCs or general to pan-VSMCs, we compared the effects of three VSMC cell lines originating from the human brain (HBVSMC), human aorta (HAVSMC), and human umbilical vein (HUVSMC) (Fig. 6a–f). At 24 h, there was no discernible difference between the HAVSMC-CM and control groups (Fig. 6a, b), but at 48 h, the number of primary neurites was increased in the HAVSMC-CM group (Fig. 6c, d). Interestingly, HUVSMC-CM decreased the primary neurite number by twofold-fold at both 24 and 48 h after plating (Fig. 6a–d).

We evaluated the number of morphologically unique unipolar-like neurons to investigate whether different types of VMSCs had an impact on particular neuronal populations. A dramatic sevenfold increase in unipolar-like neurons (the images in the right side of the Fig. 6a and c), was observed in the HUVSMC-CM-treated neurons relative to the control group (Fig. 6b, d inserts). Conversely, the secretomes of HAVSMCs and HBVSMCs did not influence the composition of unipolar-like and multipolar neuron populations (Fig. 6b, d inserts). This result implies that there is more than one way to achieve a net reduction in neurite number in response to HUVSMC-CM treatment: one approach is to promote unipolar-like cell survival while inhibiting the survival of other cells; the other approach is to inhibit the neuritogenic activity of multipolar cells.

In terms of neurite outgrowth, HAVSMC-CM had the similar promotion effect as HBVSMC-CM, while HUVSMC-CM inhibited neurite length (Fig. 6e, f). These findings showed that different VSMCs likely have different or even opposite effects on neuronal development.

### Different types of VSMCs distinctly influence neuronal firing

To test whether secretomes from different types of VSMCs distinctly influence neural circuit development, we analyzed neuronal firing in cultures treated with various VSMC-CMs. We cultured primary hippocampal neurons in 24-well plates designed for multiple electrode arrays (MEAs). Neural activity across the MEA was evaluated four times over 21 days of culture at DIV 4, 7, 10, and 14. Meanwhile, neuronal densities in each well were determined by NeuN staining at DIV 21.

Raster plots of synchronized burst recordings from representative wells showed that the HBVSMC- and HASMC-CM treatments enhanced synchronized bursts (magenta vertical bars), which represented neural connectivity (Fig. 6g). Weighted mean firing rates and burst numbers from individual active electrodes were elevated in neurons receiving HBVSMC- and HAVSMC-CMs compared to those recorded from control neurons, but this pattern was not observed in those receiving HUVSMC-CM (Fig. 6h, i).

These results were attributed to the combined enhancement of neural connectivity and neuronal survival rates in response to different VSMC-CMs. Indeed, neuron density calculations based on NeuN^+^ cells for each well following MEA assays showed that consistent with the results of high-content microscopy (Fig. 3d), HBVSMC- and HAVSMC-CMs increased neuron numbers, whereas HUVSMC-CM showed a decreasing effect (Fig. 6j). However, HUVSMC-CM treated neural network activity was equivalent to that of the control group, indicating that the population of neurons that survived produced more spikes and bursts (Fig. 6h–j).

HAVSMC-CM had a stronger effect on neural connectivity than HBVSMC-CM (Fig. 6k), despite producing a lower neuron density than HBVSMC-CM (Fig. 6j). Thus, VSMCs from the brain appeared to be the most effective in protecting neurons, whereas VSMCs from veins seemed to show an adverse effect on multipolar cells. Surprisingly, the aorta-derived VSMC cell line showed the greatest impact on neural connectivity. These human cell line results together implied that VSMCs from different vascular segments (arterial versus venular) or different organs carry differential protein signals to direct neural development.

Since ECM proteins were identified as the common molecules that are likely to influence neuronal development (Fig. 5d–h), we next focused on analyzing the common and the differential ECM proteins that are produced by HBVSMCs, MBVSMCs, HAVSMCs, and HUVSMCs. To this end, we obtained four secretomes from these four VSMC populations and have their analyzed results shown in a Venn diagram (Additional File 8: Fig. S4a). We found that there were 4 ECM proteins that were exclusively detected in HAVSMC-CM, 15 in HBVSMCM, 16 in HUVSMC-CM, and 54 in MBVSMC (Additional file 8: Fig. S4a, b).

Interestingly, among the 16 proteins found in HUVSMC-CM, *CHI3L1*, is known as a prognostic biomarker in the early stages of multiple sclerosis (MS) [32] and (ALS) [33]. Patients with high cerebrospinal fluid CHI3L1 levels have an increased risk for the development of neurological disability [32, 33]. More interestingly, the recent in vitro study also demonstrated that *CHI3L1* was neurotoxic in primary cultured neurons which induced a significant neurite length retraction and significantly reduced neuronal survival at 48 h [34]. In addition to *CHI3L1*, *COL14A1* [35] and *TPM1* [36] have also been reported to be involved in neurodegeneration [35] and aging [36]. These findings likely explain why although HUVMSC-secertome have so many ECM proteins in common with other secretomes (Additional file 8: Fig. S4c), it still showed negative effects on neurons.

Notably, among four ECM proteins that were exclusively detected in the secretome of HAVSMC, *ACAN* (Aggrecan), is the first characterized ECM protein functioning in perineuronal nets to direct extracellular matrix-mediated neuronal plasticity. These findings may directly explain why the secretome of HAVSMC profoundly increased the neural firing rate and why the secreteome of HUVSMC had detrimental effects. Together these findings suggest different organ-derived VSMCs have distinct effects on neuron development, depending on secreting distinct ECM proteins.

## Discussion

This study highlights the multifaceted instructional roles of brain VSMCs in the developing CNS beyond their classically known oxygen supply function. Here, we show that VSMC-secreted proteins play a profound role in neural circuit development processes, such as neurite initiation, neuronal survival, and circuit connectivity (Summary data in Additional file 7: Table S4). Furthermore, we show that brain VSMCs accelerate neuron maturation by positively shifting gene expression profiles. Dual analyses of the proteomics of the donor VSMC secretome and the transcriptomics of recipient neurons showed that extracellular matrix-receptor signaling is the overlapping pathway that predicts regulatory function during neural development. The secretomes of HBVSMCs, HAVSMCs, and HUVSMCs differed significantly in their regulation of neuritogenesis, survival, and later neuronal circuit connectivity. Together, our data indicate that the secretomes of VSMCs from different organs or different vasculature segments differ significantly in terms of bioactivity related to the regulation of neural development.

To our knowledge, the present study is the only report of mural smooth muscle cells instructing CNS neuronal development via secretion, although they are typically recognized as contractile cells that regulate blood flow. Thus, this study advances the neurovascular intercellular communication field by proposing that VSMCs are fundamental players in the production of angioneurins in the CNS. VSMCs belong to a small population among brain cells, accounting for less than 0.3% of cells. Their essential contractility function in the blood circulation may overshadow their alternate functions, for instance, serving as a secretory system. Thus, the involvement of these cells in neuron development has long been overlooked.

This study is the first report that neuron survival depends on VSMC bioactive secretion. Previous studies have reported that primary aortic VSMCs protect PNS superior cervical ganglion neurons from death in a coculture system without exogenous NGF, which is normally indispensable [37]. The present research largely excluded the involvement of direct physical cell‒cell contacts, but diffusible secreted proteins from VSMCs can directly protect CNS neurons. Thus, we generalized the concept of the pleiotropic function of VSMCs to the CNS.

A recent human single-cell RNA sequencing study revealed that selective cell death of mural SMCs and pericytes that belong to the M-type, which refers to the cell type secreting ECM, appears to be a newly identified risk factor for developing Alzheimer’s disease [38]. Consistently, our dual omics findings highlighting ECM pathways support this notion. Again, there are a number of lines of evidence showing that defective pial basal lamina composition, one kind of ECM protein, results in radial glia with impaired anchorage and aberrant neuronal migration [39]. Furthermore, collagen IV deficiencies, which perturb the cerebrovascular ECM, cause disease [40]. Therefore, our study implies that ECM-secreted by brain VSMCs may be involved in a range of CNS diseases, including small-vessel diseases such as cerebral autosomal dominant arteriopathy with subcortical infarcts and leukoencephalopathy (CADASIL) and cerebral autosomal recessive arteriopathy with subcortical infarcts and leukoencephalopathy (CARASIL).

The current descriptive study requires further mechanistic research with genetic disruption of one or several ECM proteins that are secreted by VSMCs to directly confirm the essential function of VSMC ECM proteins in brain development. While it is a very interesting finding that HUVSMC-CM from HBVSMCs and HAVSMCs has distinct effects on neural development, future research is needed to determine whether these effects are attributed to different organ sources (the umbilicus versus the brain and heart) or to different vessel segment sources (vein versus artery).

Together, our results provide new insights into the contribution of vascular smooth muscle to the development of neural connectivity and the prevention of neuronal death during development, revealing regulatory functions of secreted vascular components in early neuron morphogenesis, which are tightly involved in neuron survival and subsequent circuit connections.

## Supplementary Information

**Additional file 1: Figure S1.** The procedure for preparing and characterization of VSMC-CMs. **a** Flow chart for preparing VSMC-conditioned medium. **b** Bright-field images of HBVSMCs after treatment with neuronal medium for 24, 48, and 72 h. **c** Relative cell viability curve of HBVSMCs in **b**.

**Additional file 2: Table S1.** pH and osmotic pressure detection of conditioned medium.

**Additional file 3: Figure S2.** HBVSMC-CM increases dendrite numbers. **a** Representative image of hippocampal neurons after a 72-h treatment with HBVMSC-CM. Anti-Tuj1 (green), anti-Map2 (red), Hoechst (blue). **b** Bar graph of the average total dendrite length of neurons according to MAP2 immunoreactive signals. Data are presented as the mean ± SD.

**Additional file 4: Figure S3.** Sorting and confirming the purity of primary pial VSMCs from *SMACreER:Ai14*. **a** Representative FACS plots of mixed cells from the pia of pup brains with approximately 6% tdTomato^+^ VSMCs before sorting (left) and 99.17% tdTomato^+^ VSMCs after sorting (right). **b** Bright-field image, fluorescent image, and merged image of the sorted tdTomato + VSMCs (left panel). Quantification of the percentage of tdTomato^+^ VSMCs (right panel). **c** Agarose gel electrophoresis RT‒PCR products using species-specific PCR primer sets for a-SMA and 18S rRNA (top panel). Semiquantitative analysis of RT‒PCR products (bottom panel).

**Additional file 5: Table S2.** The differentially expressed genes (DEGs) in neuron cultured with VSMC-CM compared with control.

**Additional file 6: Table S3.** Common secreted proteins in HBVSMC-CM and MBVSMC-CM.

**Additional file 7: Table S4.** Data summary of statistical results for neurite number, average total neurite length and neuron density.

**Additional file 8: Figure S4.** ECM proteins in different SMC secretomes. **a** Veen diagraph to show the differential distribution of ECM proteins from different secretomes. Proteins with a Sum PEP Score > 2 were chosen and filtered with the The Extracellular Matrix Interaction Database in MatrixDB. **b** ECM proteins included exclusively in different secretomes were represented. **c** Common ECM proteins in every secretomes were represented.

## Data Availability

The datasets used and/or analyzed during the current study are available from the corresponding author on reasonable request.

## Abbreviations

HBVSMC: Human brain vascular smooth muscle cell
MBVSMC: Mouse brain vascular smooth muscle cell
CM: Conditioned medium
ECM: Extracellular matrix
PNS: Peripheral nervous system
CNS: Central nervous system
MEA: Multielectrode array
GO: Gene ontology
KEGG: Kyoto encyclopedia of genes and genome
DIV: Days in vitro
ROI: Region of interest
