# Supplementary figures and images for: Proteins secreted by brain arteriolar smooth muscle cells are instructive for neural development
Source: Mol Brain. 2022 Nov 30;15:97. doi: 10.1186/s13041-022-00983-y (PMC9710182; doi:10.1186/s13041-022-00983-y)

## Slide 1
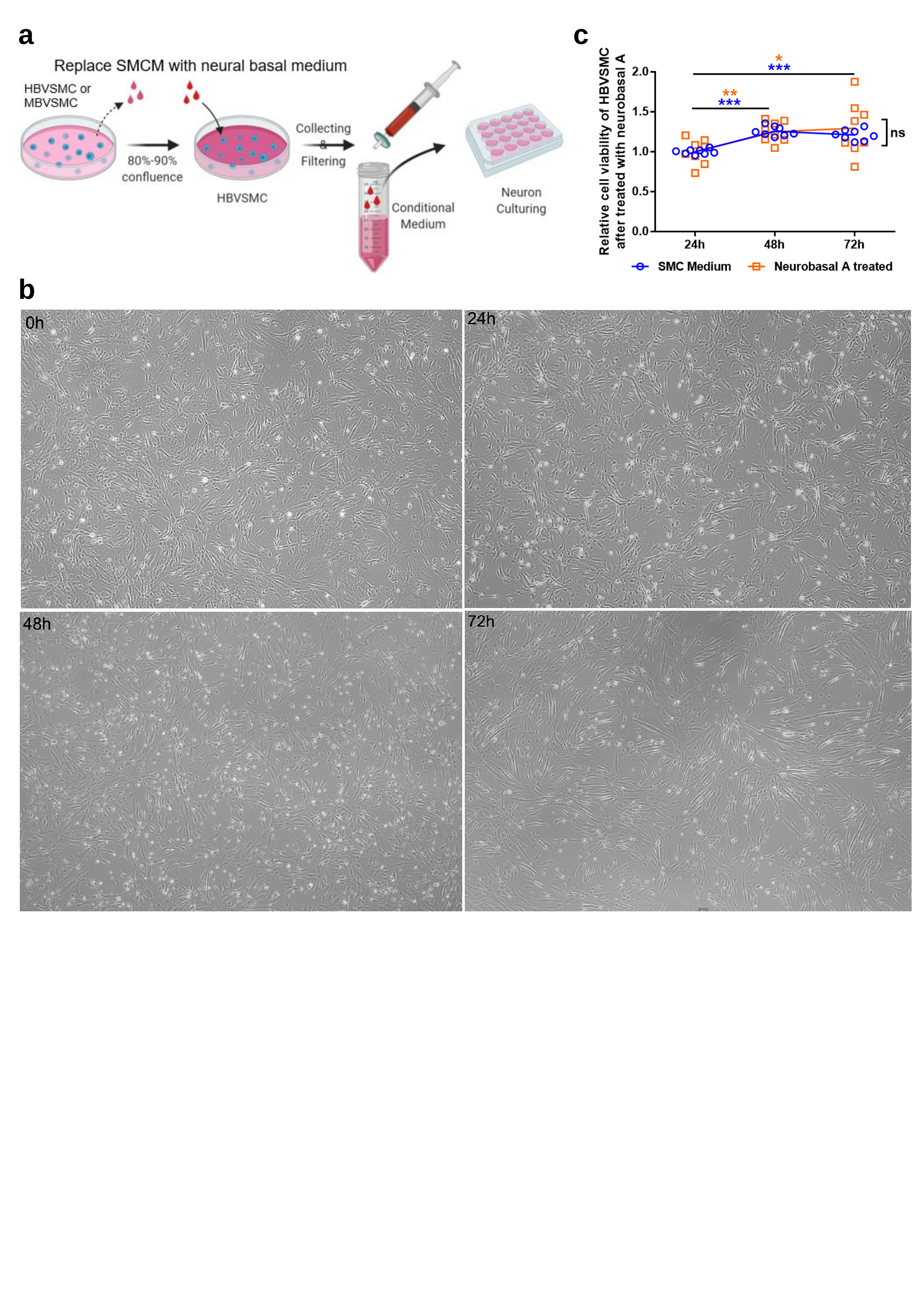

a
c
b

Supplement: Supplementary file 1 — Additional file 1: Figure S1. The procedure for preparing and characterization of VSMC-CMs. a Flow chart for preparing VSMC-conditioned medium. b Bright-field images of HBVSMCs after treatment with neuronal medium for 24, 48, and 72 h. c Relative cell viability curve of HBVSMCs in b. [file 13041_2022_983_MOESM1_ESM.pptx]

## Slide 1
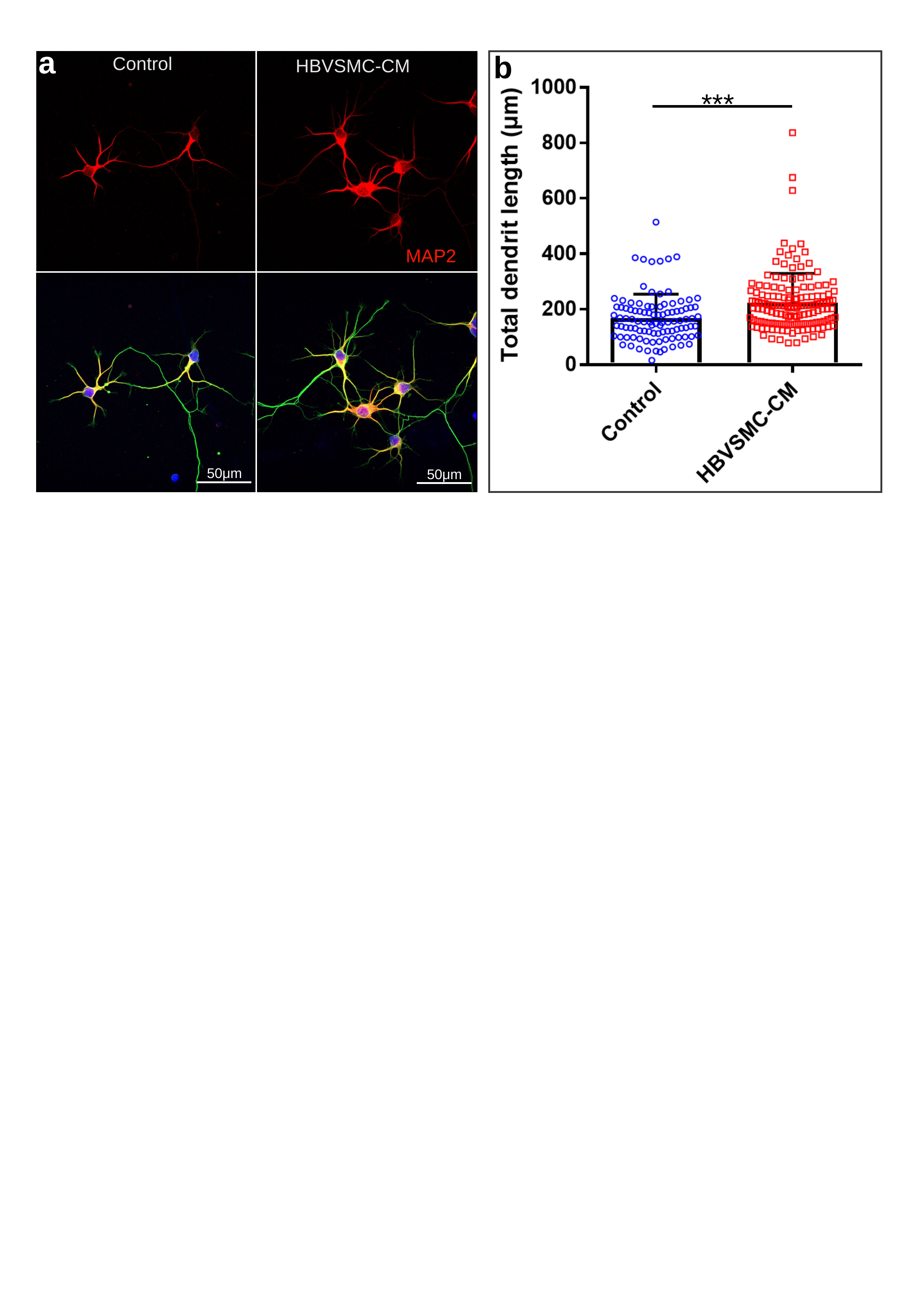

a
b
Control
HBVSMC-CM
***
MAP2
50μm
50μm

Supplement: Supplementary file 3 — Additional file 3: Figure S2. HBVSMC-CM increases dendrite numbers. a Representative image of hippocampal neurons after a 72-h treatment with HBVMSC-CM. Anti-Tuj1 (green), anti-Map2 (red), Hoechst (blue). b Bar graph of the average total dendrite length of neurons according to MAP2 immunoreactive signals. Data are presented as the mean ± SD. [file 13041_2022_983_MOESM3_ESM.pptx]

## Slide 1
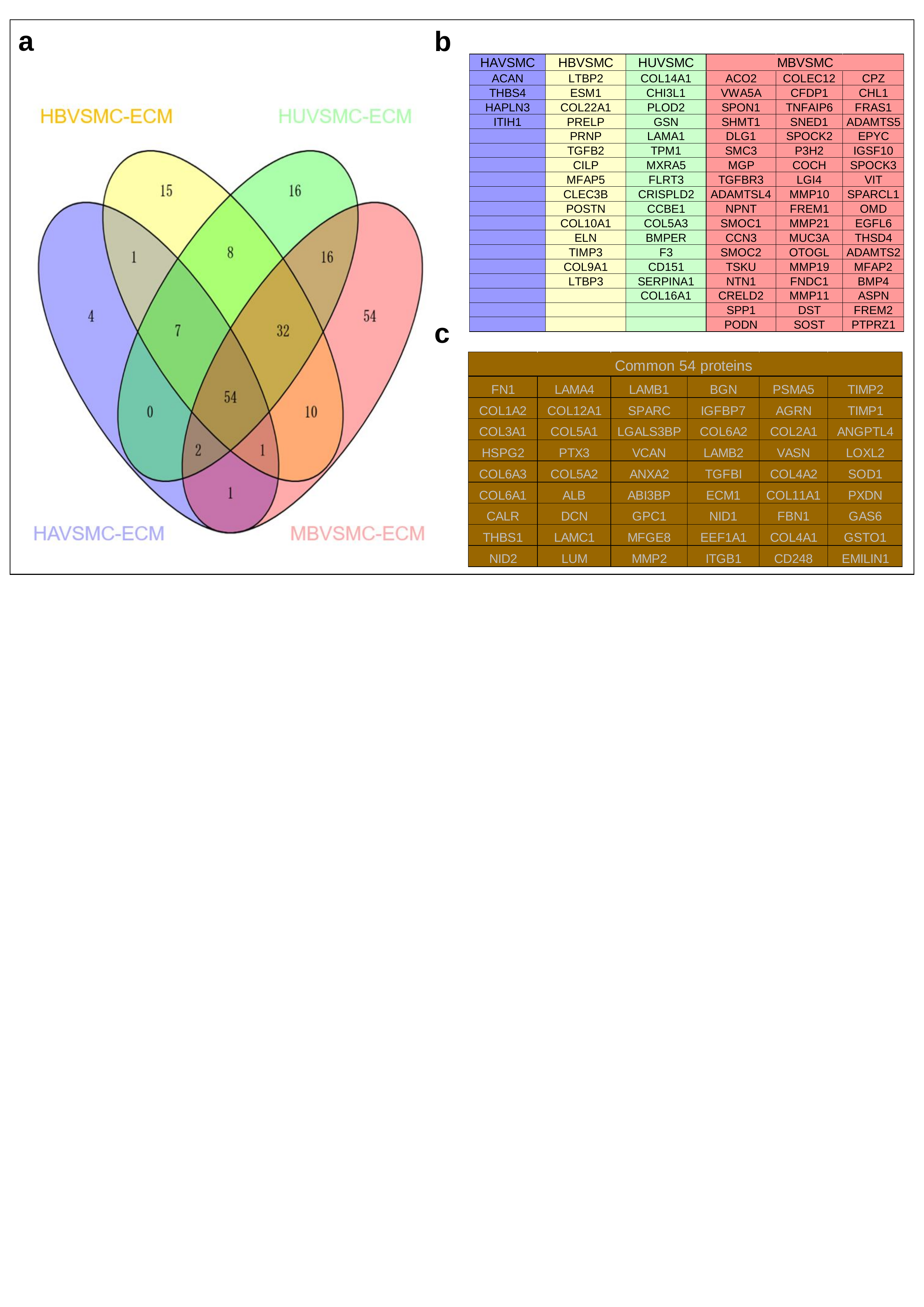

a
b
c

Supplement: Supplementary file 8 — Additional file 8: Figure S4. ECM proteins in different SMC secretomes. a Veen diagraph to show the differential distribution of ECM proteins from different secretomes. Proteins with a Sum PEP Score > 2 were chosen and filtered with the The Extracellular Matrix Interaction Database in MatrixDB. b ECM proteins included exclusively in different secretomes were represented. c Common ECM proteins in every secretomes were represented. [file 13041_2022_983_MOESM8_ESM.pptx]
