# Supplementary material for: Proteins secreted by brain arteriolar smooth muscle cells are instructive for neural development
Source: Mol Brain. 2022 Nov 30;15:97. doi: 10.1186/s13041-022-00983-y (PMC9710182; doi:10.1186/s13041-022-00983-y)
Supplement: Supplementary file 2 — Additional file 2: Table S1. pH and osmotic pressure detection of conditioned medium. [file 13041_2022_983_MOESM2_ESM.docx]

Table S1. pH and osmotic pressure of conditioned medium

|  | pH | Osmotic pressure (mmol/kg) |
| --- | --- | --- |
| Salin1 |  | 287 |
| Salin2 |  | 286 |
| Neuron medium_1 | 7.38 | 259 |
| Neuron medium_2 | 7.37 | 257 |
| HBVSMC-CM_1 | 7.36 | 265 |
| HBVSMC-CM_2 | 7.36 | 267 |
