# Supplementary material for: Proteins secreted by brain arteriolar smooth muscle cells are instructive for neural development
Source: Mol Brain. 2022 Nov 30;15:97. doi: 10.1186/s13041-022-00983-y (PMC9710182; doi:10.1186/s13041-022-00983-y)
Supplement: Supplementary file 4 — Additional file 4: Figure S3. Sorting and confirming the purity of primary pial VSMCs from SMACreER:Ai14. a Representative FACS plots of mixed cells from the pia of pup brains with approximately 6% tdTomato+ VSMCs before sorting (left) and 99.17% tdTomato+ VSMCs after sorting (right). b Bright-field image, fluorescent image, and merged image of the sorted tdTomato + VSMCs (left panel). Quantification of the percentage of tdTomato+ VSMCs (right panel). c Agarose gel electrophoresis RT‒PCR products using species-specific PCR primer sets for a-SMA and 18S rRNA (top panel). Semiquantitative analysis of RT‒PCR products (bottom panel). [file 13041_2022_983_MOESM4_ESM.pptx]

## Slide 1
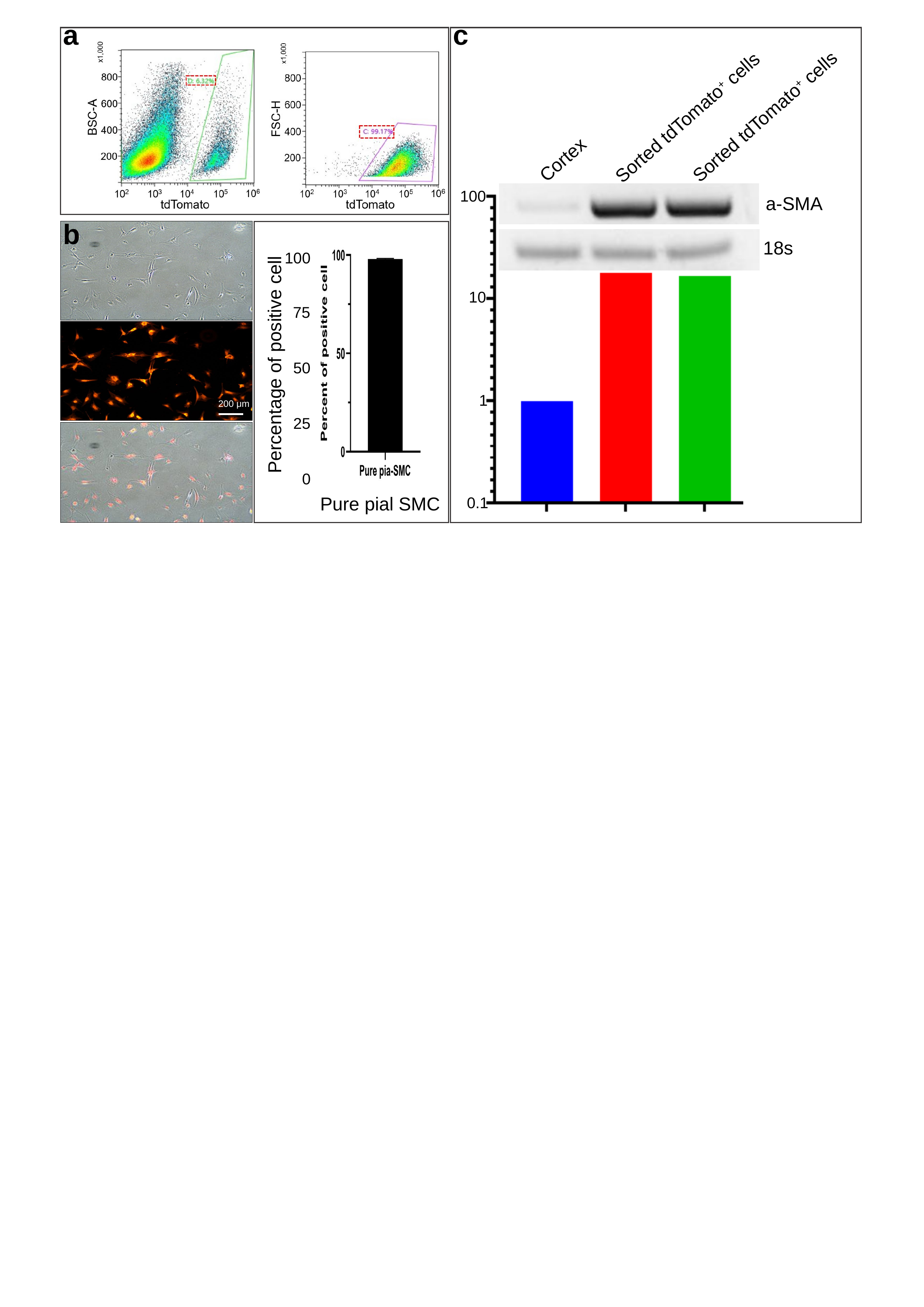

a
c
Sorted tdTomato+ cells
Sorted tdTomato+ cells
Cortex
100
a-SMA
b
18s
100
10
75
Percentage of positive cell
50
1
200 μm
25
0
Pure pial SMC
0.1
