# Supplementary material for: Proteins secreted by brain arteriolar smooth muscle cells are instructive for neural development
Source: Mol Brain. 2022 Nov 30;15:97. doi: 10.1186/s13041-022-00983-y (PMC9710182; doi:10.1186/s13041-022-00983-y)
Supplement: Supplementary file 7 — Additional file 7: Table S4. Data summary of statistical results for neurite number, average total neurite length and neuron density. [file 13041_2022_983_MOESM7_ESM.docx]

**Table S4. Data summary of neurite number, average total neurite length and neuron density**

|  |  | Culture time | Neurite number  (mean ± SD) | Neurite number  (Fold change) | Neurite length (μm)  (mean ± SD) | Neurite length  (Fold change) | Neuron density  (/mm^2^) | Neuron density  (Fold change) |
| --- | --- | --- | --- | --- | --- | --- | --- | --- |
| Group 1 | Control | 36h | 3.86±1.72 | 1 | 130.3±70.83 | 1 | — |  |
|  |  | 48h | 4.91±1.85 | 1 | 211.0±106.00 | 1 | 141.8±44.65 | 1 |
|  |  | 72h | 5.29±1.52 | 1 | 436.4±187.80 | 1 | — |  |
|  | MBVSMC-CM | 36h | 5.36±2.04 | 1.39 | 225.9±95.57 | 1.73 | — |  |
|  |  | 48h | 5.85±1.82 | 1.19 | 359.7±155.40 | 1.7 | 371.1±110.6 | 2.62 |
|  |  | 72h | 7.20±1.63 | 1.36 | 633.4±242.60 | 1.45 | — |  |
| Group 2 | Control | 24h | 3.67±1.49 | 1 | 91.18±43.55 | 1 |  |  |
|  |  | 48h | 4.67±1.66 | 1 | 247.2±113.60 | 1 | 41.0±19.0 | 1 |
|  |  | 72h | 5.33±1.41 | 1 | 505.7±174.40 | 1 |  |  |
|  | HBVSMC-CM | 24h | 4.27±1.82 | 1.16 | 156.5±69.91 | 1.72 | — |  |
|  |  | 48h | 5.97±2.13 | 1.28 | 304.4±132.10 | 1.23 | 67.7±27.3 | 1.65^###^ |
|  |  | 72h | 7.69±2.16 | 1.44 | 578.0±199.20 | 1.14 | — |  |
| Group 3 | Control | 24h | 3.53±1.37 | 1 | 83.98±40.55 | 1 |  |  |
|  |  | 48h | 4.06±1.27 | 1 | 147.9±60.75 | 1 | 110.8±28.67 | 1 |
|  | HAVSMC-CM | 24h | 3.89±1.66 | 1.1 | 94.98±43.71 | 1.13 | — |  |
|  |  | 48h | 5.51±1.93 | 1.35 | 227.0±96.16 | 1.53 | 133.4±29.16 | 1.21^###^ |
|  | HBVSMC-CM | 24h | 4.21±1.80 | 1.19 | 161.4±66.57 | 1.92 |  |  |
|  |  | 48h | 5.89±2.14 | 1.45 | 212.7±93.54 | 1.44 | 139.4±54.08 | 1.26^###^ |
|  | HUVSMC-CM | 24h | 2.17±1.08 | 0.61 | 52.01±26.30 | 0.62 | — |  |
|  |  | 48h | 3.03±1.37 | 0.75 | 63.74±35.53 | 0.43 | 77.8±30.2 | 0.70 |

###:<0.001, indicate the significant different of normalized neuron density fold in HBVAMC, HAVSMC compared with that in MBVSMC.
